# Impact of digital health literacy on health-related quality of life in Chinese community-dwelling older adults: the mediating effect of health-promoting lifestyle

**DOI:** 10.3389/fpubh.2023.1200722

**Published:** 2023-06-21

**Authors:** Siqi Liu, Ya Lu, Dan Wang, Xiaochong He, Wei Ren, Dehui Kong, Yu Luo

**Affiliations:** School of Nursing, Army Medical University (Third Military Medical University), Chongqing, China

**Keywords:** health-related quality of life, digital health literacy, electronic health literacy, eHealth literacy, health-promoting lifestyle, mediating effect, older adults

## Abstract

**Background:**

In the context of aging and digitalization, the development and application of digital health can help meet the growing health needs of older adults. Improving digital health literacy of older adults may be an effective way to alleviate the shortage of public health resources and improve their health-related quality of life (HRQoL). However, the impact of digital health literacy on HRQoL in older adults and the underlying mechanism remain unclear. This study intends to explore whether digital health literacy has an effect on HRQoL in community-dwelling older adults, and whether health-promoting lifestyle plays a mediating role between digital health literacy and HRQoL, while providing a theoretical basis for the scientific construction of HRQoL intervention programs for older adults.

**Methods:**

The cross-sectional study was conducted in Chongqing, China from September 2020 to April 2021. 572 community-dwelling older adults were surveyed by stratified sampling. Data on sociodemographic characteristics, digital health literacy, health-promoting lifestyle and HRQoL were collected. Univariate analysis was used to compare the differences in HRQoL among community-dwelling older adults with different sociodemographic characteristics. Pearson correlation analysis was used to explore the correlation between digital health literacy, health-promoting lifestyle and HRQoL. SPSS PROCESS macro was used to examine the mediating effect of health-promoting lifestyle between digital health literacy and HRQoL.

**Results:**

The mean score of HRQoL was 97.97 (SD 11.45). Univariate analysis showed that there were statistically significant differences in HRQoL among community-dwelling older adults with different gender, age, educational level, marital status, and monthly household income *per capita* (*p* < 0.05). There were positive correlations between digital health literacy, health-promoting lifestyle and HRQoL, with correlation coefficients ranging from 0.416 to 0.706 (*p* < 0.001). Digital health literacy was positively associated with HRQoL (*β* = 0.210, *p* < 0.001), and health-promoting lifestyle mediated the relationship between digital health literacy and HRQoL, with an indirect effect of 0.175 (95% Bootstrap CI 0.135–0.214).

**Conclusion:**

Digital health literacy can affect HRQoL through the mediating effect of health-promoting lifestyle. It is suggested that relevant management institutions, communities and families should strengthen the cultivation of the digital health literacy of older adults, promote their development of health-promoting lifestyle, and ultimately improve HRQoL.

## 1. Introduction

Global aging is progressing at an unprecedented rate and will further accelerate in the coming decades (1), directly resulting in a significant increase in the demand for public health services and medical expenditures (2). Properly solving the problem of population aging and improving the health-related quality of life (HRQoL) of older adults have become important issues of global concern. HRQoL is a key component of healthy aging (3), and refers to an individual’s subjective feelings and overall satisfaction with respect to their physical function, mental state, and social abilities (4, 5). The results of systematic reviews confirmed that HRQoL of older adults is closely related to sociodemographic factors, social well-being, health status and health behaviors (6, 7). Among them, factors such as sociodemographic characteristics and chronic disease conditions are difficult to change. In order to effectively improve HRQoL of older adults, it is necessary for health professionals to seek modifiable factors as new targets to implement interventions.

The rapid advances in information and communications technology (ICT) contribute to the increasing innovation, and upgrading of health service mode and the emergence of digital health offers a new idea for improving the HRQoL of older adults. Especially during the COVID-19 pandemic, the vulnerable older adults are advised to stay at home and try to avoid unnecessary trips by the public health authority (8). Most of the supply to meet their needs for health services has to shift to online (9). Digital health enables low-cost, high-quality and prompt health care services for senior citizens and plays an important role in maintaining their physical and mental health (10). Digital health literacy, also known as electronic health literacy or eHealth literacy, reflects the ability to make use of the digital health services effectively, which is defined as an individual’s ability to search, understand and evaluate the health information on digital media, actively engage in the exchange and interactions of health information, and use the acquired information for health management and health problem-solving (11, 12). An increasing body of evidence suggests that the digital health literacy-related interventions can significantly improve the health of older adults and promote healthy aging (10, 13). However, there is limited empirical research exploring the relationship between digital health literacy and HRQoL, merely with inconsistent conclusions. According to the longitudinal and cross-sectional studies of Iranian older adults, digital health literacy and HRQoL were significantly correlated (14, 15). However, the survey on American older adults showed no significant correlation between digital health literacy and HRQoL (16). The latest systematic review also underlined that the impact of digital health literacy on the HRQoL of older adults is unclear and there is still a lack of study into the underlying mechanism of effect (17).

The Integrative Model of eHealth Use (IMeHU) suggests that people with better digital health literacy have higher motivation and ability to use the Internet to obtain health information, more active online health behaviors, easier to master more health knowledge and form positive health beliefs and health behaviors (18). The results of the systematic review also proved that the use of digital health tools can stimulate positive health behaviors in older adults and promote the development of health-promoting lifestyle (19), suggesting that health-promoting lifestyle may be a potential mediating factor. Health-promoting lifestyle refers to actions that individuals take the initiative to pursue, which could benefit their health, including health responsibility, nutrition, physical activity, interpersonal relations, stress management, and spiritual growth (20). A previous study on Chinese college students found that health-promoting lifestyle played an important mediating role between health literacy and HRQoL (21). Digital health literacy is an extension of health literacy in the field of digital health (22). The mediating effect of health-promoting lifestyle between digital health literacy and HRQoL in older adults may also be established, but it remains to be confirmed by empirical research.

**Figure 1:**
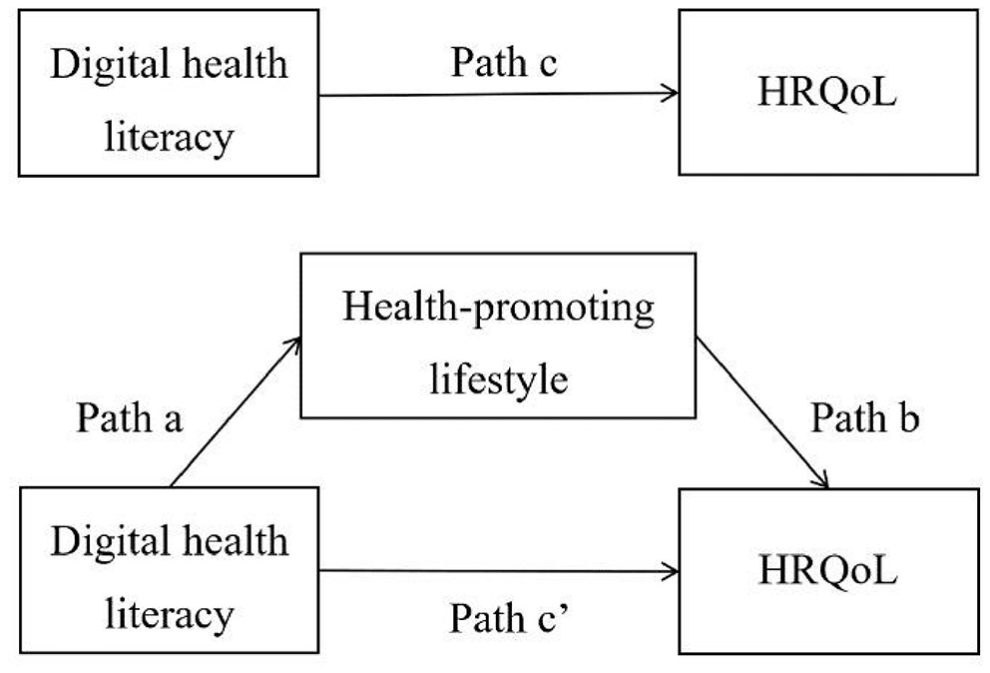
The hypothesised model.

As the country with the largest older adults population in the world (23), China has 191 million people over the age of 65 (24), where the aging situation is extremely severe. In the last decade, Chinese government has been committed to actively promoting the adoption of ICT into the healthcare domain (25, 26), as well as strengthening the smart senior care industry (27). Taking Chinese community-dwelling older adults as an example, the current study discusses the effective ways to improve HRQoL of older adults from the unique perspective of digital health literacy, which can provide new ideas and methods for developing health promotion programs to enhance HRQoL in the future. Therefore, the objective of this study was to explore the relationship between digital health literacy and HRQoL of older adults and factors that mediate the association, and we developed the following priori hypotheses (Figure 1): (1) Digital health literacy will be positively correlated with HRQoL; (2) Health-promoting lifestyle will mediate the relationship between digital health literacy and HRQoL.

## 2. Methods

### 2.1. Participants

This cross-sectional study was carried out in the main urban areas of Chongqing, China from September 2020 to April 2021. The sample size was estimated using the formula for epidemiology study in estimating the mean of continuous outcome: $n={\left(\frac{Z_{1-\alpha /2}\times \sigma }{\delta }\right)}^{2}$ (28). According to a previous study, the standard deviation of HRQoL in Chinese older adults was 18.29 (29). Thus, we set σ as 18.29 in this study. Using α = 0.05, Z_1-α/2_ = 1.96, δ = 2.00, the calculated sample size was 321. Multistage stratified sampling method was used for sample selection. Firstly, according to the statistics of Chongqing Statistics Bureau in 2019 (30), we categorized 9 administrative districts in main urban areas of Chongqing into three levels according to gross domestic product (GDP) *per capita*, and 1 administrative district was randomly selected from each category. Then 2 ~ 5 communities were selected from each of the selected administrative districts, and a total of 10 communities were used as sample sites for the study. Lastly, older adults who met the inclusion criteria were recruited from the selected communities for a questionnaire survey. The inclusion criteria were permanent residents in main urban areas of Chongqing and aged 65 years or older. The exclusion criteria were mental illness diagnosis, a definite diagnosis of severe physical illness, and declining to participate.

Research group members with standardized training served as investigators, explaining the purpose of the research to participants, and distributing paper questionnaires after obtaining written and oral informed consent. Questionnaires were filled out by participants themselves. Those who had difficulty in filling in the forms themselves were assisted by an investigator to read the questions one by one and record their answers. Once each participant completed the questionnaire, the investigators immediately checked on-site and asked the participant to complete the questionnaire if there were any omissions. In the end, A total of 600 questionnaires were distributed and 572 valid questionnaires were obtained, with an effective recovery rate of 95.33%. Our research was ethically approved by the Ethics Committee of Army Medical University/Third Military Medical University (approval number 2020–012-02). And survey administration was conducted according to the Declaration of Helsinki.

### 2.2. Measures

The questionnaire contained questions regarding sociodemographic characteristics, digital health literacy, health-promoting lifestyle, and HRQoL.

#### 2.2.1. Assessment of sociodemographic characteristics

A self-designed sociodemographic information questionnaire was used to collect data on participants’ gender, age (65–69, 70–74, 75–79 and ≥ 80 years), education level (primary school or below, junior high school, senior high school, college or above), marital status (with spouse or without spouse), and monthly household income per capital (<1,000, 1,000–2,999, 3,000–4,999, ≥5,000).

#### 2.2.2. Digital health literacy assessment scale for community-dwelling older adults (DHLAS)

Digital Health Literacy Assessment Scale for community-dwelling older adults was developed by our research group in the previous research (see Supplementary material for complete scale) (31). It was the first original digital health literacy assessment tool targeted for Chinese older adults, based on the digital health background and digital devices usage characteristics of older adults in China, and proven to be with good internal consistency (0.941), spilt-half reliability (0.889), test–retest reliability (0.941), content validity (0.967), criterion validity (0.938) and construct validity. The scale included 3 dimensions of digital health information acquisition and evaluation ability (9 items), digital health information interaction ability (3 items), and digital health information application ability (3 items), with a total of 15 items. Answers were based on a 5-point Likert scale ranging from 1 (“strongly disagree”) to 5 (“strongly agree”). The total score was 15–75 points. Higher scores indicate a higher level of digital health literacy. Cronbach’s α coefficient for the total scale in this study was 0.959.

#### 2.2.3. Health promoting lifestyle profile-II, revise (HPLP-IIR)

Health Promoting Lifestyle Profile (HPLP) was developed by Walker et al. (32) to measure individual health promotion behaviors. This study employed the Chinese revised version by Cao et al. (33), with a total of 40 items, including 6 dimensions of physical activity (8 items), health responsibility (11 items), stress management (5 items), nutrition (6 items), interpersonal relations (5 items) and spiritual growth (5 items). The scale used a 4-point Likert scale ranging from 1 (“never”) to 4 (“always”). The total score was 40–160 points. Higher scores indicate a healthier lifestyle. The instrument has been validated for use in Chinese older adults, the reliability of which has been reported with a Cronbach’s α coefficient 0.82 for total scale (34). In this study, Cronbach’s α coefficient was 0.926.

#### 2.2.4. 12-item short form health survey (SF-12)

12-item short form health survey (SF-12), the abbreviated version of the 36-item short form health survey (SF-36), was developed by the health Institute of New England Medical Center to assess HRQoL (35). The scale is consisted of 12 items, including 8 dimensions, namely general health (GH), physical functioning (PF), role physical (RP), bodily pain (BP), role emotional (RE), vitality (VT), mental health (MH) and social functioning (SF). The first four dimensions were combined into the physical component summary (PCS), while the last four dimensions were combined into the mental component summary (MCS). PCS and MCS were scored with a mean of 50 and a SD of 10, ranging from 0 to 100 in the general population (35, 36), reflecting individual’s physical and mental health status, respectively. According to the score criteria, the total score was transformed into standard scores, with higher score indicating better HRQoL. Chinese version SF-12 has been verified to have good reliability and validity when applying to the health status evaluation of Chinese community-dwelling older adults, with Cronbach’s α coefficient of 0.910 (37). The Cronbach’s α coefficient in this study was 0.884.

### 2.3. Data analysis

Data was analysed with SPSS 23.0. Continuous variables conforming to normal distribution (such as digital health literacy, health-promoting lifestyle, and HRQoL, etc.) were described by means ± standard deviation (SD), and categorical variables (such as gender, education level, etc.) were described by *N* (%). Independent sample t test and one-way analysis of variance (ANOVA) were used to compare the differences of HRQoL among community-dwelling older adults with different sociodemographic characteristics. Pearson correlation analysis was used to explore the correlation between digital health literacy, health-promoting lifestyle and HRQoL of older adults.

The hypothesized mediation model (Figure 1) was examined via the PROCESS macro in SPSS (38), which is widely used to test the mediating effects in current studies (9, 39). We used the digital health literacy as the prediction variable, health-promoting lifestyle as the mediator variable, and HRQoL as the outcome variable. Meanwhile, sociodemographic factors, including gender, age, education level, marital status, and monthly household income *per capita*l, were used as covariates in the analyses to overcome the potential confounding effects. The coefficient c’ was the direct effect of digital health literacy on HRQoL. Digital health literacy might also indirectly influence HRQoL through health-promoting lifestyle, the effects of which were captured by the produce of coefficients a and b (a × b). Coefficient c was the total effect of digital health literacy on HRQoL, namely c’ + a × b. Point estimates were based on 5,000 bootstrap samples, and 95% confidence intervals (CI) were constructed. An indirect effect was considered significant if the CI did not contain zero. All statistical tests were two-tailed and statistical significance for all analysis was set at 0.05.

## 3. Results

### 3.1. Sociodemographic characteristics and HRQoL status of samples

This study included 572 community-dwelling older adults with a mean age of 70.93 (SD 5.51) years, ranging from 65 to 88 years. More than half of participants only had junior high school education or below (388/572, 67.83%). Among all participants, the mean score of HRQoL was 97.97 (SD 11.45), in which the mean score of PCS was 44.70 (SD 9.45), and the MCS was 53.26 (SD 6.81). The results of univariate analysis indicated that there were statistically significant differences in HRQoL among older adults with different gender, age, education level, marital status, and monthly household income *per capita* (*p* < 0.05). Female, 70 years old and above, without spouse, primary school education or below, monthly household income *per capita* below 1,000 yuan, are the characteristics older adults with which have a lower level of HRQoL compared with other groups. For further details, see Table 1.

**Table 1:** The status of HRQoL by different sociodemographic characteristics (*N* = 572).

Characteristics	*n* (%)	HRQoL			
		Mean ± SD	*t* or *F*	*p* value	*Post hoc*
Gender			2.232	0.026	
Male	273 (47.73%)	99.08 ± 11.28			
Female	299 (52.27%)	96.95 ± 11.53			
Age (years)			10.853	<0.001	
65-69^a^	283 (49.48%)	100.46 ± 10.04			a > b, c, d
70-74^b^	144 (25.17%)	96.44 ± 11.70			
75-79^c^	92 (16.08%)	95.81 ± 12.09			
≥80^d^	53 (9.27%)	92.57 ± 13.53			
Education level			12.214	<0.001	
Primary school or below^a^	202 (35.31%)	94.36 ± 12.38			a < b, c, d
Junior high school^b^	186 (32.52%)	98.84 ± 10.56			
Senior high school^c^	114 (19.93%)	101.27 ± 10.75			
College and above^d^	70 (12.24%)	100.68 ± 9.18			
Marital status			2.959	0.003	
With spouse	444 (77.62%)	98.80 ± 10.87			
Without spouse	128 (22.38%)	95.09 ± 12.92			
Monthly household income *per capita* (RMB)			12.359	<0.001	
<1000^a^	42 (7.34%)	90.50 ± 11.78			a < b, c, d
1,000-2999^b^	216 (37.76%)	96.66 ± 12.15			b < d
3,000-4999^c^	242 (42.31%)	99.03 ± 10.67			c < d
≥5000^d^	72 (12.59%)	102.68 ± 8.72			

The lowercase letters a,b,c and d represent different groups of the characteristics of different age, education level and monthly household income *per capita*.

### 3.2. Correlations between digital health literacy, health-promoting lifestyle, and HRQoL

Pearson’s r correlation analysis indicated that digital health literacy and health-promoting lifestyle were significantly positively associated with HRQoL (*r* = 0.416, *p* < 0.001; *r* = 0.507, *p* < 0.001). Meanwhile, digital health literacy was also significantly positively associated with health-promoting lifestyle (*r* = 0.706, *p* < 0.001) (Table 2). The significant correlation between the three variables suggested that the relationship between the variables could be further analysed and explained by establishing a model for regression analysis.

**Table 2:** Correlations (r) between digital health literacy, health-promoting lifestyle, and HRQoL (*N* = 572).

Variables	Mean ± SD	1	2	3
1.Digital health literacy	37.10 ± 18.65	1	–	–
2.Health-promoting lifestyle	109.53 ± 16.64	0.706^***^	1	–
3.HRQoL	97.97 ± 11.45	0.416^***^	0.507^***^	1

^***^p < 0.001.

### 3.3. Mediation test for health-promoting lifestyle

**Figure 2:**
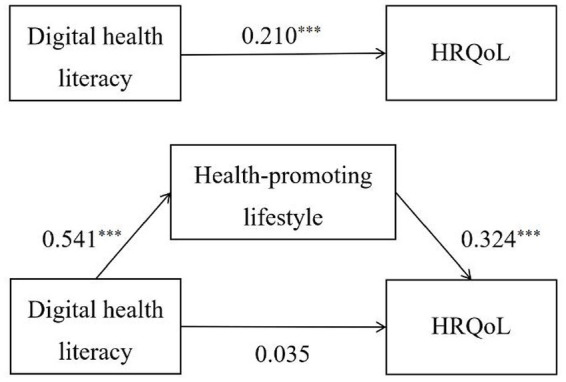
Health-promoting lifestyle mediated the association of digital health literacy and HRQoL. ***p < 0.001.

Tables 3, 4 and Figure 2 presented the mediation analysis result based on the PROCESS macro. After controlling sociodemographic factors, the total effect of digital health literacy on HRQoL was significant (c = 0.210, *p* < 0.001). Then putting the mediation variable of health-promoting lifestyle into the model, the direct effect was statistically non-significant (c’ = 0.035, *p* > 0.05). In addition, digital health literacy had a significant predictive effect on health-promoting lifestyle (a = 0.541, *p* < 0.001), and the predictive effect of health-promoting lifestyle on HRQoL was also significant (b = 0.324, *p* < 0.001). We further generated 5,000 bootstrapping samples from the original dataset by random sampling to assess the size of the indirect effect. The results indicated that this indirect effect (a × b) was 0.175, with a 95% CI from 0.135 to 0.214. The bootstrap 95% CI did not conclude zero, revealing that the mediating effect was statistically significant. Thus, health-promoting lifestyle mediated the association between digital health literacy and HRQoL, and the model accounted for 30.3% of the total variance of HRQoL.

**Table 3:** Multivariate linear regression analysis for the association among digital health literacy, health-promoting lifestyle, and HRQoL (*N* = 572).

Variables	Model 1 (HRQoL)			Model 2 (Health-promoting lifestyle)			Model 3 (HRQoL)			B (SE)	t	*p* value	B (SE)	t	*p* value	B (SE)	t	*p* value
Gender	−1.613 (0.905)	−1.782	0.075	5.411 (0.997)	5.428	<0.001	−3.364 (0.869)	−3.874	<0.001
Age	−0.279 (0.086)	−3.259	0.001	0.038 (0.094)	0.401	0.689	−0.291 (0.080)	−3.637	<0.001
Education level	−0.309 (0.549)	−0.563	0.574	1.768 (0.605)	2.926	0.004	−0.882 (0.517)	−1.704	0.089
Marital status	−0.681 (1.117)	−0.610	0.542	−2.464 (1.230)	−2.004	0.046	0.117 (1.048)	0.112	0.911
Monthly household income *per capita*	1.295 (0.669)	1.935	0.054	2.180 (0.737)	2.958	0.003	0.589 (0.631)	0.934	0.351
Digital health literacy	0.210 (0.029)	7.280	<0.001	0.541 (0.032)	17.006	<0.001	0.035 (0.033)	1.061	0.289
Health-promoting lifestyle							0.324 (0.036)	9.057	<0.001
*R*^2^	0.202			0.542			0.303		
*F*	23.811 (*p* < 0.001)			111.455 (*p* < 0.001)			35.056 (*p* < 0.001)		

**Table 4:** Mediation analyses of health-promoting lifestyle in the association between digital health literacy and HRQoL.

	Effect	95% Bootstrap CI	Bootstrap SE
Total effect (c)	0.210	(0.152, 0.269)	0.030
Direct effect (c’)	0.035	(−0.028, 0.099)	0.032
Indirect effect (a × b)	0.175	(0.135, 0.214)	0.021

## 4. Discussion

In the context of aging and digitalization, this study taking Chinese community-dwelling older adults as example, explored and confirmed the impact of digital health literacy on HRQoL of older adults and the mediating effect of health-promoting lifestyle in the relationship. It has important reference value for future theoretical and practical research on improving HRQoL for older adults.

Our findings revealed a significant difference in the HRQoL score of older adults by gender, age, education level, marital status, and monthly household income *per capita*, which was consistent with the results of previous studies (40–42). These identified non-modifiable factors can be used to determine which individuals are at risk of poor HRQoL, and to identify the vulnerable population that health professionals need to target. In health education, we should pay high attention to population who are female, aged 70 and older, have no partner, only received primary education or below, with a monthly household income per capita less than 1,000 yuan and deliver more health interventions to improve their HRQoL.

This study found that digital health literacy was positively correlated with HRQoL of community-dwelling older adults, which confirmed Hypothesis 1. The possible explanation is that in the context of the digital health era, higher digital health literacy means more varied health information resources and more efficient digital health services. In the daily life, more health information resources will help older adults change their wrong health awareness and health lifestyle, to build more positive health behaviors and maintain better health (43). When seeking health services, the older adults who are skilled in using digital health services will be able to manage their health in a more efficient manner and communicate with health personnels in real time so that they can be provided with more comprehensive and customized health services to help with disease treatment and management (44). During the COVID-19 pandemic, conventional offline diagnosis and treatment are restricted and the impact of digital health literacy on HRQoL has been further expanded. The older adults who lack of digital health literacy are unable to make effective use of online health information platforms, telemedicine service and other digital health services, thus it becomes extremely inconvenient for them to get daily health consultations, purchase drugs and so on (45). At the same time, faced with massive and complicated information about the pandemic, it is hard to identify the real information from the wrong one and older adults are highly susceptible to negative emotions like fear and anxiety which have a great impact on their health, both physically and mentally (46). Therefore, older adults as the vulnerable group in the Internet age are affected far more than other groups in HRQoL because of lacking digital health literacy. Health professionals should give sufficient attention to them.

This study also found that health-promoting lifestyle fully mediated the association between digital health literacy and HRQoL of community-dwelling older adults, which preliminarily elucidated the mechanism of association between digital health literacy and HRQoL, and confirmed Hypothesis 2. The mediating effect was mainly divided into two stages, namely, (1) digital health literacy had a positive effect on health-promoting lifestyle, and (2) health-promoting lifestyle had a positive effect on HRQoL. Both stages have been confirmed in previous studies as ample literature support for this study (14, 47–50). Meanwhile, it is worth emphasizing that this study adopted DHLAS based on the current context of the Internet in China and the characteristics of digital devices usage among older adults to assess digital health literacy, which is different from former universal scale survey and is a further expansion of previous research conclusions. The mediating effect of health-promoting lifestyle between digital health literacy and HRQoL can be explained by Andersen’s Behavioral Model of Health Services Use (51). This model emphasizes that individual characteristics is an antecedent variable influencing health behaviors and health outcomes. It can either directly affect health outcomes or indirectly affect health outcomes by influencing health behaviors (52). Enabling resources in the dimension of individual characteristics refers to the availability of health service resources and the ability of individuals to access health services (53). Digital health literacy can be seen as one of the key competencies. It can be inferred based on this model that older adults with higher digital health literacy are more confident in using digital health services and more active in accessing effective health resources through digital media, thereby improving their awareness of health management and adoption of science-based health management behaviors, developing more positive health-promoting lifestyle that in return improve HRQoL.

This study has important theoretical and practical implications for improving HRQoL of community-dwelling older adults. First, this study evaluates the digital health literacy of older adults with an original designed tool based on the characteristics of older adults digital health behaviours to fill the gap in the literature, expounding the relationship between digital health literacy and HRQoL of older adults, and enrich the conclusions of previous studies. Second, this study explores and confirms the important mediating effect of health-promoting lifestyle between digital health literacy and HRQoL of older adults with a new perspective. Third, this study may provide new ideas for the interventions to improve HRQoL for older adults. The findings suggest that in the future health professionals may intervene in HRQoL of older adults from the perspectives of digital health literacy and health-promoting lifestyle. On the one hand, communities should carry out normalized digital health literacy training. By building community exchange and mutual assistance platforms combined with family guidance, the digital health literacy of older adults can be jointly promoted to help them obtain and apply health information more efficiently, and promote the transformation of health behaviors. On the other hand, health professionals should strengthen daily health education to guide older adults to adopt a healthy lifestyle such as doing physical exercises and keeping a balanced diet, so as to enhance HRQoL.

This study has certain limitations. First, our research is cross-sectional, which makes it impossible to clarify the causal relationship and direction of effect among digital health literacy, health-promoting lifestyle and HRQoL. Longitudinal or intervention studies could be conducted in the future to further confirm the longitudinal association or causality between them. Second, this study was only conducted in Chongqing, China. Considering that individual digital health literacy level and HRQoL status are closely related to local economic development level, we should pay attention to the extrapolation of the research results. In future studies, the sample size should be further expanded and a large-scale cross-regional survey should be conducted to make the research results more representative. Third, this study used self-report measures, which may have some information bias.

## 5. Conclusion

This study focuses on the impact of digital health literacy on HRQoL of community-dwelling older adults and explores the mediating effect of health-promoting lifestyle in a creative way by constructing and confirming the mediation model. To some extent, this enriches the perspectives of digital health literacy study and offers an updated idea for improving HRQoL of community-dwelling older adults. Relevant regulatory authorities, communities, and families are prompted to endeavor to raise the digital health literacy of older adults, broaden access to health information and build up their ability to apply it in the future, assist with the development of health-promoting lifestyle, and help them fully enjoy the convenience of digital health to improve HRQoL and promote healthy aging.

## Data availability statement

The raw data supporting the conclusions of this article will be made available by the authors, without undue reservation.

## Ethics statement

The studies involving human participants were reviewed and approved by the ethical committees of Army Medical University/Third Military Medical University. The patients/participants provided their written informed consent to participate in this study.

## Author contributions

YuL obtained funding for the study. SL, YaL, and YuL conceived and designed the study. DW, XH, and WR coordinated the study. SL, YaL, DW, XH, and WR collected the data. SL, DK analysed and interpreted the data. SL and YaL drafted the manuscript. YuL and DK revised the manuscript. All authors contributed to the article and approved the submitted version.

## Funding

The research was supported by the National Social Science Fund of China (Grant No. 19XRK001).

## Conflict of interest

The authors declare that the research was conducted in the absence of any commercial or financial relationships that could be construed as a potential conflict of interest.
