# Supplementary material for: Impact of digital health literacy on health-related quality of life in Chinese community-dwelling older adults: the mediating effect of health-promoting lifestyle
Source: Front Public Health. 2023 Jun 21;11:1200722. doi: 10.3389/fpubh.2023.1200722 (PMC10321557; doi:10.3389/fpubh.2023.1200722)
Supplement: Supplementary file 1 [file Data_Sheet_1.DOC]

**English language translation of the original survey** (the questionnaires in the English language version are the free translations of the authors, they have not been validated for research use)

**Digital Health Literacy Assessment Scale for community-dwelling older adults**

I would like to ask you for your opinion and about your experience using the Internet for health information. For each statement, tell me which response best reflects your opinion and experience right now.（1=Strongly disagree，2=disagree，3=Undecided，4=Agree，5=Strongly agree）

| 1.I am interested in learning health knowledge or skills on the Internet. | £1 £2 £3 £4 £5 |
| --- | --- |
| 2.I will check the health information pushed on the Internet. | £1 £2 £3 £4 £5 |
| 3.I will search the Internet initiatively for the health information I need. | £1 £2 £3 £4 £5 |
| 4.I can understand the content of Internet health information. | £1 £2 £3 £4 £5 |
| 5.I can tell if Internet health information is correct. | £1 £2 £3 £4 £5 |
| 6.I judge the reliability of Internet health information by consulting others. | £1 £2 £3 £4 £5 |
| 7.I can filter out the content I need from the Internet health information. | £1 £2 £3 £4 £5 |
| 8.Even if it is credible, high-quality Internet health information, I will carefully consider whether it is appropriate for my personal situation. | £1 £2 £3 £4 £5 |
| 9.When I find and share health information on the Internet, I will pay attention to protecting the privacy of myself and others. | £1 £2 £3 £4 £5 |
| 10.I can share or comment on health information resources on the Internet (such as forwarding or commenting on health information in Wechat, etc.). | £1 £2 £3 £4 £5 |
| 11.I can participate in health forum or online discussion (such as the health discussion in Wechat group, QQ group, etc.) | £1 £2 £3 £4 £5 |
| 12.I can participate in online polls or surveys on health topics. | £1 £2 £3 £4 £5 |
| 13.I can consult online through the Internet to provide doctors with the information they need for diagnosis (such as symptom description, past medical records, health indicators, etc.) | £1 £2 £3 £4 £5 |
| 14.I can use the relevant functions of health websites or online health platforms (such as appointment registration, online payment, access to eHealth records, etc.) | £1 £2 £3 £4 £5 |
| 15.I can use electronic devices to record and manage personal health indicators (such as blood pressure, heart rate, weight, etc.) | £1 £2 £3 £4 £5 |
